# Parkinson’s disease and cancer: a systematic review and meta-analysis on the influence of lifestyle habits, genetic variants, and gender

**DOI:** 10.18632/aging.203932

**Published:** 2022-03-05

**Authors:** Joon Yan Selene Lee, Jing Han Ng, Seyed Ehsan Saffari, Eng-King Tan

**Affiliations:** 1 Department of Neuroscience and Behavioural Disorders Programme, Duke-NUS Medical School, Singapore; 2 Department of Neurology, National Neuroscience Institute, Singapore

**Keywords:** Parkinson’s disease, cancer, LRRK2, meta-analysis, systematic review

## Abstract

Purpose: The relationship between Parkinson’s disease (PD) and cancer has been debated. Gender and genetic influences on cancer development in PD is unclear.

Methods: Using QUOROM guidelines, we conducted a systematic review and meta-analysis on potential clinical and genetic factors influencing the PD and subsequent cancer relationship. English articles published in PubMed, Web of Science, and SCOPUS from 2010 to 30 August 2020 were considered for suitability.

Results: Of 46 studies identified, fourteen satisfied the inclusion criteria and were further analysed. Unadjusted risk ratios (RR) and 95% confidence intervals were computed to determine the PD and cancer relationship. PD patients have decreased subsequent cancer risks (RR = 0.87, 95% CI = 0.81–0.93), reduced risks of colon, rectal, and colorectal cancer (RR = 0.77, 95% CI = 0.63–0.94), lung cancer (RR = 0.62, 95% CI = 0.48–0.80), and increased brain cancer (R = 1.48, 95% CI = 1.02–2.13) and melanoma risk (R = 1.76, 95% CI = 1.23–2.50). Compared to idiopathic PD, LRRK2-G2019S carriers had increased general cancer risks (RR = 1.26, 95% CI = 1.09–1.46), particularly brain (RR = 2.41, 95% CI = 1.06–5.50), breast (RR = 2.57, 95% CI = 1.19–5.58), colon (RR = 1.83, 95% CI = 1.13–2.99), and haematological cancers (RR = 2.05, 95% CI = 1.07–3.92). Female PD patients have decreased general cancer risks compared to male PD patients in this analysis (RR = 0.83, 95% CI = 0.69–0.98).

Conclusion: PD patients have reduced risks of colon, rectal, colorectal cancer and lung cancers and increased risks of brain cancer and melanoma. LRRK2-G2019S carriers have increased cancer risks, particularly brain, breast, colon and blood cancers. Female gender was associated with reduced risks. The role of ethnicity, comorbidities, and lifestyle habits on PD patients’ subsequent cancer risk should be further investigated.

## INTRODUCTION

Parkinson's disease (PD) is a neurodegenerative disease characterized by resting tremors, bradykinesia, and rigidity [1]. The burden of disease to PD patients, their caregivers, and society is high. PD has been associated with an increase in disability-adjusted life years (one of the leading causes of years lived with disability [2]).

Intriguingly, studies have identified a relationship between cancer development following PD diagnosis. Several studies uncovered a positive relationship between PD and subsequent melanoma [3, 4], while some found a null relationship [5, 6]. Other studies also noted melanoma development after levodopa use, the standard PD pharmacological therapy [7].

Clearly, existing studies investigating the link between PD and subsequent melanoma have reached different conclusions. Furthermore, PD was associated with increased risks of breast [4], non-melanocytic [8], and brain cancer [9], but decreased prostate, bladder, and colorectal cancer risks [10]. Risk factors including gender, gene variants implicated in PD pathogenesis, and lifestyle habits like smoking, were also found to have effects on cancer risk following PD diagnosis, further adding to the debate.

Cancer is characterized by aberrant and uncontrolled proliferation [11], directly opposing PD pathogenesis. However, like PD, cancer is crippling due to the physical decline, high mortality, treatment effects, and psychological trauma involved [12]. This makes it more pertinent to interrogate the link between PD and subsequent cancer risk, to identify and treat both diseases early, and more importantly identify factors (such as gender, genetic predisposition, and lifestyle habits) that may influence the association between PD and cancer. Collectively, these efforts can reduce the overall disease burden associated with both pathologies.

To address these gaps in knowledge, we conduct a systematic review and meta-analysis (including case-control and cohort studies published between 1 January 2010 and 30 August 2020) to investigate the association of specific cancers with PD and the possible role of lifestyle, gender and genetic risk factors.

## RESULTS

### Included studies

**Figure 1:**
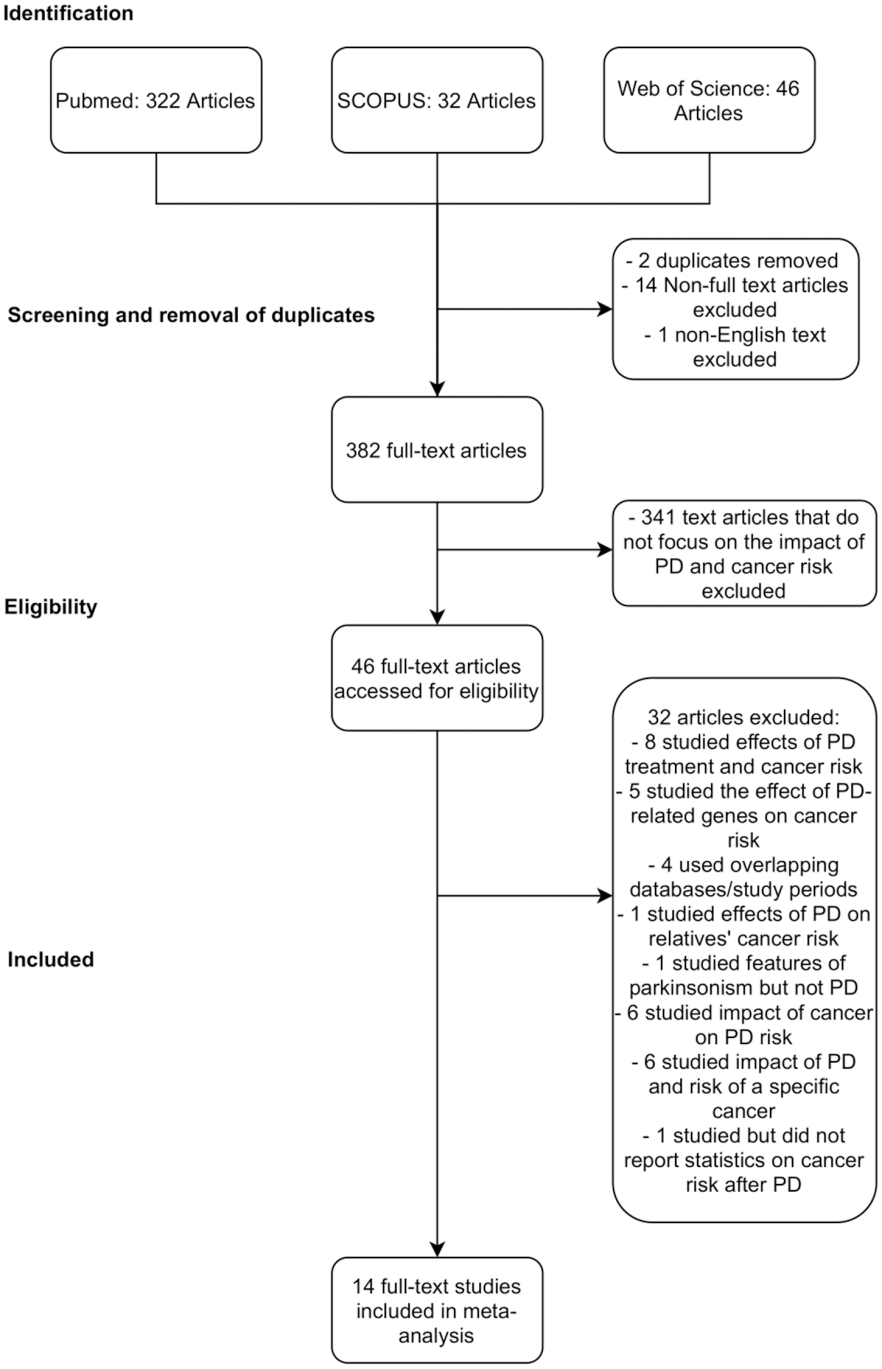
PRISMA chart detailing database search procedure and exclusion criteria.

Fourteen studies were included in this meta-analysis, conducted in accordance with the Quality of Reporting of Meta-analyses (QUOROM) guidelines (Figure 1, Supplementary Table 1).

The details of the 14 studies are provided in Table 1. Reasons for excluding studies investigating PD’s impact on subsequent cancer risk, despite meeting the eligibility criteria, are detailed in Supplementary Table 2.

**Table 1:** Characteristics of all studies included in the meta-analysis.

**No.**	**Author**	**Study design**	**Country**	**Sample size**	**Females (%)**	**Mean age (SD)**	**Adjustment**	**Cancer (s) reported**
1	Lin, 2015	Cohort	Taiwan	62023 PD patients 124046 non-PD controls	94458 (50.7%)	NR	Sex, age	Cancer in general, brain, melanoma, kidney, liver, uterus (women), oesophagus, skin, prostate (men), gallbladder, lymphoma/leukaemia, stomach, bladder, lung, pancreas, colorectal, cervical (women), breast (women), thyroid, ovary (women)
2	Fois, 2010	Cohort	UK	4355 PD patients Compared to general population	2205 (50.6%)	NR	Sex, age in 5y bands, time period (years), district of residence	Cancer in general, oral cavity, pharynx, lip, larynx, oesophageal, stomach, colon, rectum, pancreas, lung, breast, cervix, ovary, uterus, prostate, kidney, bladder, malignant melanoma, other skin cancer, malignant brain, bone, lymphoma, non-Hodgkin's lymphoma, multiple myeloma, leukaemia, lymphoid leukaemia, myeloid leukaemia, benign brain
3	Peretz, 2016	Cohort	Israel	7125 PD patients Compared to general population	3297 (46.3%)	71.1 (10.6)	Age, chronological year, sex	Cancer in general, breast (women), colon, CNS, kidney, leukaemia, lung, lymphoma, melanoma, ovary, pancreas, prostate (men), rectum, thyroid
4	Park, 2019	Cohort	South Korea	52009 PD patients 260045 non-PD controls	184776 (59.2%)	71 (10)	Diabetes mellitus, hypertension, dyslipidaemia, income status	Cancer in general, oral cavity and pharyngeal, laryngeal, oesophageal, gastric, colorectal, liver, pancreatic, biliary, lung, renal, bladder, thyroid, leukaemia, lymphoma, multiple myeloma, skin, breast (women), uterine cervical (women), uterine corpus (women), ovarian (women), prostate (men), testicular (men)
5	Lo, 2010	Cohort	USA	692 PD patients 761 non-PD controls	544 (37.4%)	65.9 (12.1)	Age, sex, cigarette smoking (pack years), alcohol consumption (number of drinks per month), BMI, eye colour	Cancer in general, smoking-related cancer, non-smoking related cancer, lung, bladder, breast (women), prostate (men), colorectal, melanoma
6	Liat, 2014	Cohort	UK	219194 PD patients 9015614 non-PD controls	43%	NR	NR	Cancer in general, bladder, bone, brain, breast (women), cervix (women), colon, upper GI, kidney, larynx, myeloid leukaemia, lymphoid leukaemia, liver, lung, Hodgkin's lymphoma, non-Hodgkin's lymphoma, malignant melanoma, multiple myeloma, nasopharynx, meninges, oesophageal, ovary (women), pancreas, prostate, rectum, salivary gland, non-melanoma skin cancer, stomach, testis (men), thyroid, uterus corpus (women)
7	Rugbjerg, 2012	Cohort	Denmark	20343 PD patients Compared to general population	9631 (47.3%)	72.7	NR	Cancer in general, malignant melanoma, non-melanoma skin, breast (women), larynx, lung, urinary bladder, ovary, fallopian tube and bread ligament (women), colorectal, prostate (men), non-Hodgkin lymphoma, corpus uteri (women), brain, multiple myeloma, lymphatic leukaemia, unspecified
8	Wirdefeldt, 2014	Cohort	Sweden	11786 PD patients 58930 non-PD controls	27906 (39.5%)	62.5 (9.2)	Education level	Cancer in general, mouth, oesophageal, stomach, liver, pancreas, nose and nasal sinuses, larynx, trachea, bronchus, lung and pleura, cervix uteri (women), kidney and urinary organs, small intestine, peritoneum, mediastinum, breast (women), prostate (men), testis (men), malignant melanoma of skin, skin (excluding melanoma), endocrine glands, bone, connective tissue or muscle, nervous system, colon, rectum, anus, lymphoma, corpus uteri (women), ovary (women), male genital organs other than prostate and testis, thyroid gland, multiple myeloma, lymphatic leukaemia, unspecified
9a	Becker, 2010	Cohort	UK	2993 PD patients 3003 non-PD controls	NR	NR	NR	Cancer in general, lung, larynx, pharynx, buccal cavity, stomach, urinary tract, oesophageal, pancreas, breast (women), colorectal, prostate (men), lymphoma/leukaemia, female reproductive organs, CNS, liver, gallbladder, thyroid gland, unspecified
9b^1^	Becker, 2010	Case-control	UK	1118 PD patients 1212 non-PD controls	NR	NR	NR	Cancer in general, lung, larynx, pharynx, buccal cavity, stomach, urinary tract, oesophageal, pancreas, breast (women), colorectal, prostate (men), lymphoma/leukaemia, female reproductive organs, CNS, liver, gallbladder, thyroid gland, unspecified
10	Agalliu, 2019	Case-control	Europe, Israel, USA	712 PD patients 218 non-PD controls	419 (45.1%)	66.9 (10.9)	Age, sex, Ashkenazi Jews ethnicity (fixed effect) and study centre (random effect), smoking status, BMI	Cancer in general, skin cancer, melanoma, lung cancer, bladder cancer, breast (women), ovarian (women), prostate (men), colon, kidney/renal, haematologic/lymphoma, meningioma
11	Ruiz-Martínez, 2014	Case-control	Spain	637 PD patients 176 non-PD controls	415 (51.0%)	71.2 (12.0)	Age	Cancer in general, melanoma, lung, bladder, colon, kidney, breast (women), ovarian (women), prostate (men), hormonal, haematologic, meningioma, unspecified
12	Freedman, 2015	Case-control	USA	6994 PD patients 972822 non-PD controls	445388 (45.5%)	NR (Median age = 74 years)	Age, race, sex, number of doctors’ visits, cancer registry area and selection years	Cancer in general, oral cavity, oesophageal, stomach, colon, rectum, pancreas, larynx, lung and bronchus, melanoma, breast (women), cervix (women), uterus (women), ovary (women), prostate (men), urinary bladder, kidney/renal pelvis, thyroid, leukaemia
13	Tacik, 2016	Case-control	USA	971 PD patients 478 non-PD controls	840 (58.0%)	NR (median age = 67 years)	Age and sex (except for sex-specific cancers - breast, prostate, ovarian, uterine, testicular) No adjustment - For cancers with <10 patients	Cancer in general, breast (women), colon, leukaemia, lymphoma, prostate (males), bladder, pancreatic, melanoma, nonmelanoma skin cancer, any skin cancer, ovarian (women), lung, brain, stomach, bile duct, uterine (women), oesophageal, liver, thyroid, bone, kidney, testicular (men)
14	Shalaby, 2016	Case-control	USA	108 PD patients 124 non-PD controls	127 (54.7%)	71.4 (7.94)	Liberal adjusted (any cancer): Number of prescription medications, age in years, Caucasian race, Cumulative Illness Rating Scale Score Liberal adjusted (melanoma): Number of prescription medications Liberal adjusted (integumentary cancers): Number of prescription medications, years since last hospitalisation Conservative adjusted (all cancers, melanoma, integumentary cancers): Similar to unadjusted model - Include variables associated with both the cancer and the diagnosis	Cancer in general, basal cell, squamous integumentary, brain, squamous mesodermal, breast (women), lymphoma, lymphoma, myeloma, leukaemia, oral cavity/pharynx, uterine (women), ovarian (women), prostate (men), urinary/bladder, kidney, thyroid, gastric, colon, liver, pancreas, unspecified

Abbreviations: PD: Parkinson’s Disease; NR: Not reported; BMI: Body mass index; GI: Gastrointestinal.

Subgroup analysis comparing cancer risk after PD diagnosis in LRRK2-G2019S mutation carriers and idiopathic PD patients included six studies. Three studies were included in the primary analysis [13–15] while three [16–18] were identified during the initial database search. The characteristics of these six studies are summarized in Supplementary Table 3.

Similarly, analysis comparing the cancer risk after PD diagnosis between female and male PD patients included four studies. Three studies [4, 19, 20] were included in the primary analysis, while one [6] was identified during the initial database search. The characteristics of the four studies are summarized in Supplementary Table 4.

### General cancer risks and heterogeneity of studies

**Figure 2:**
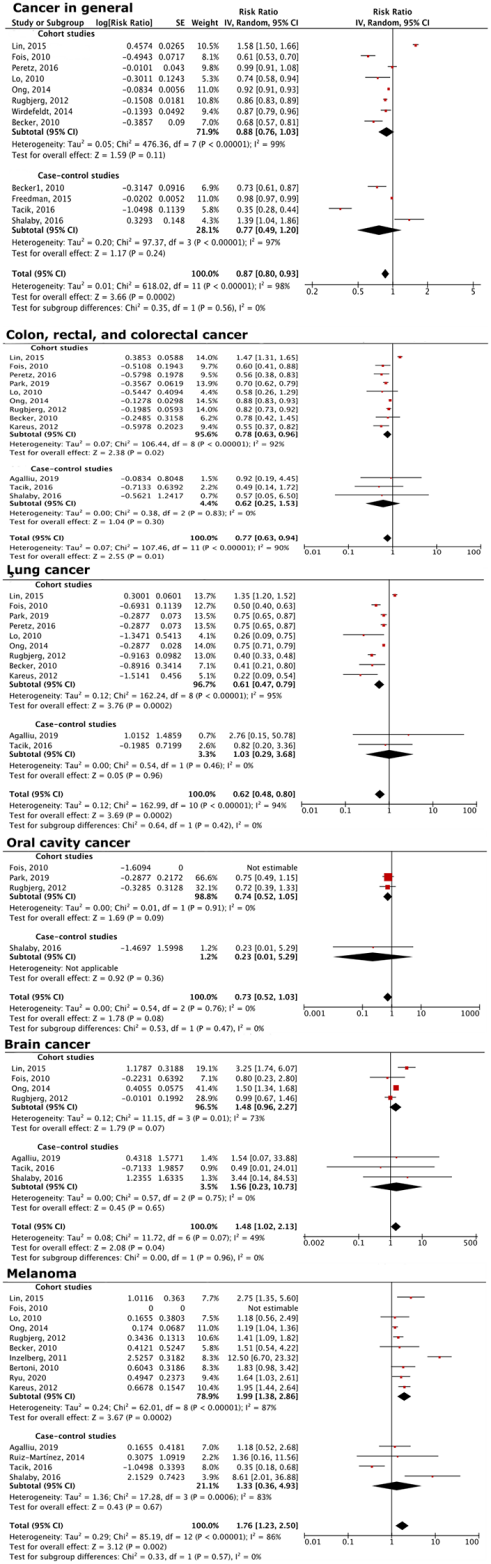
**Forest plot of the association between PD and overall cancer risk, as well as that of specific cancers.** PD patients had decreased overall cancer risks, and decreased risks of colon, rectal, colorectal, lung, oral cavity, brain cancers, and melanoma, compared to the general population.

In this study, PD was associated with a reduced relative risk of subsequent cancer development (RR = 0.87, 95% CI = 0.81–0.93; data not shown). This association held true even after sensitivity analysis (RR = 0.87, 95% CI = 0.80–0.93; Figure 2). This is consistent with previous studies indicating an inverse relationship between PD and cancer.

### Risk of specific cancers

Subgroup analysis revealed that PD is associated with a decrease in smoking-related cancers, including colon, rectal, and colorectal cancer (RR = 0.77, 95% CI = 0.63–0.94), lung cancer (RR = 0.62, 95% CI = 0.48–0.80), and oral cancers (RR = 0.73, 95% CI = 0.52–1.03; Figure 2), even though statistical significance was not reached for oral cancer.

The subgroup analysis also showed that PD was associated with an increased risk of brain cancers (RR = 1.48, 95% CI = 1.02–2.13) and melanoma (RR = 1.76, 95% CI = 1.23–2.50; Figure 2). These conclusions are consistent with earlier findings [9, 21, 22].

### Increased cancer risks in LRRK2-G2019S PD patients

**Figure 3:**
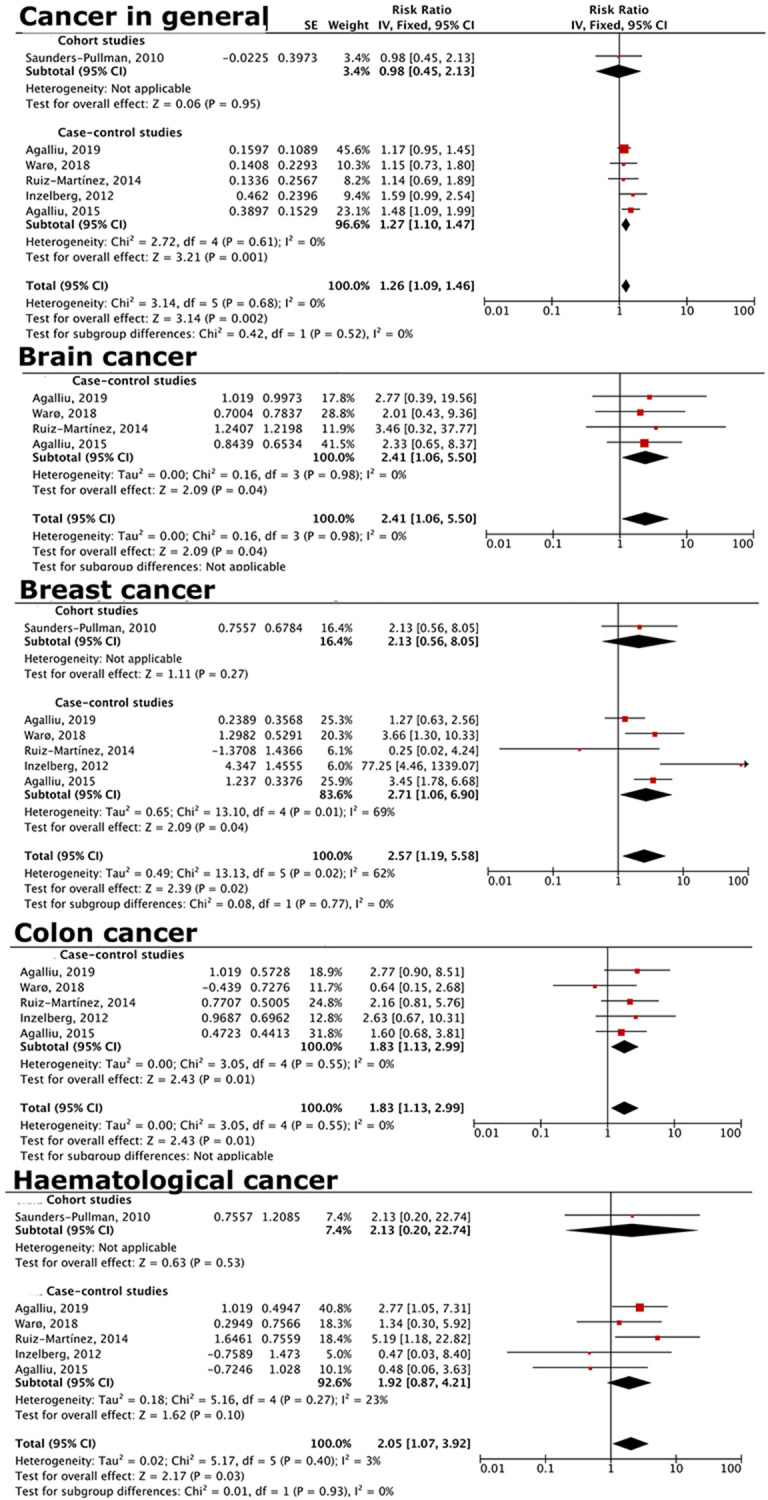
**Forest plot comparing risks of cancer in general and specific cancers for LRRK2-PD vs. idiopathic PD patients.** LRRK2-PD patients had higher risk of overall cancer, as well as brain, breast, colon, and hematological cancers.

A comparison of subsequent cancer risk in LRRK2-G2019S PD and idiopathic PD patients revealed that LRRK2-G2019S PD patients had an increased risk of cancer in general (RR = 1.26, 95% CI = 1.09–1.46; Figure 3). This increase was particularly seen in brain (RR = 2.41, 95% CI = 1.06–5.50), breast (RR = 2.57, 95% CI = 1.19–5.58), colon (RR = 1.83, 95% CI = 1.13–2.99), and hematological cancers (RR = 2.05, 95% CI = 1.07–3.92; Figure 3).

### Decreased cancer risks in female PD patients

**Figure 4:**
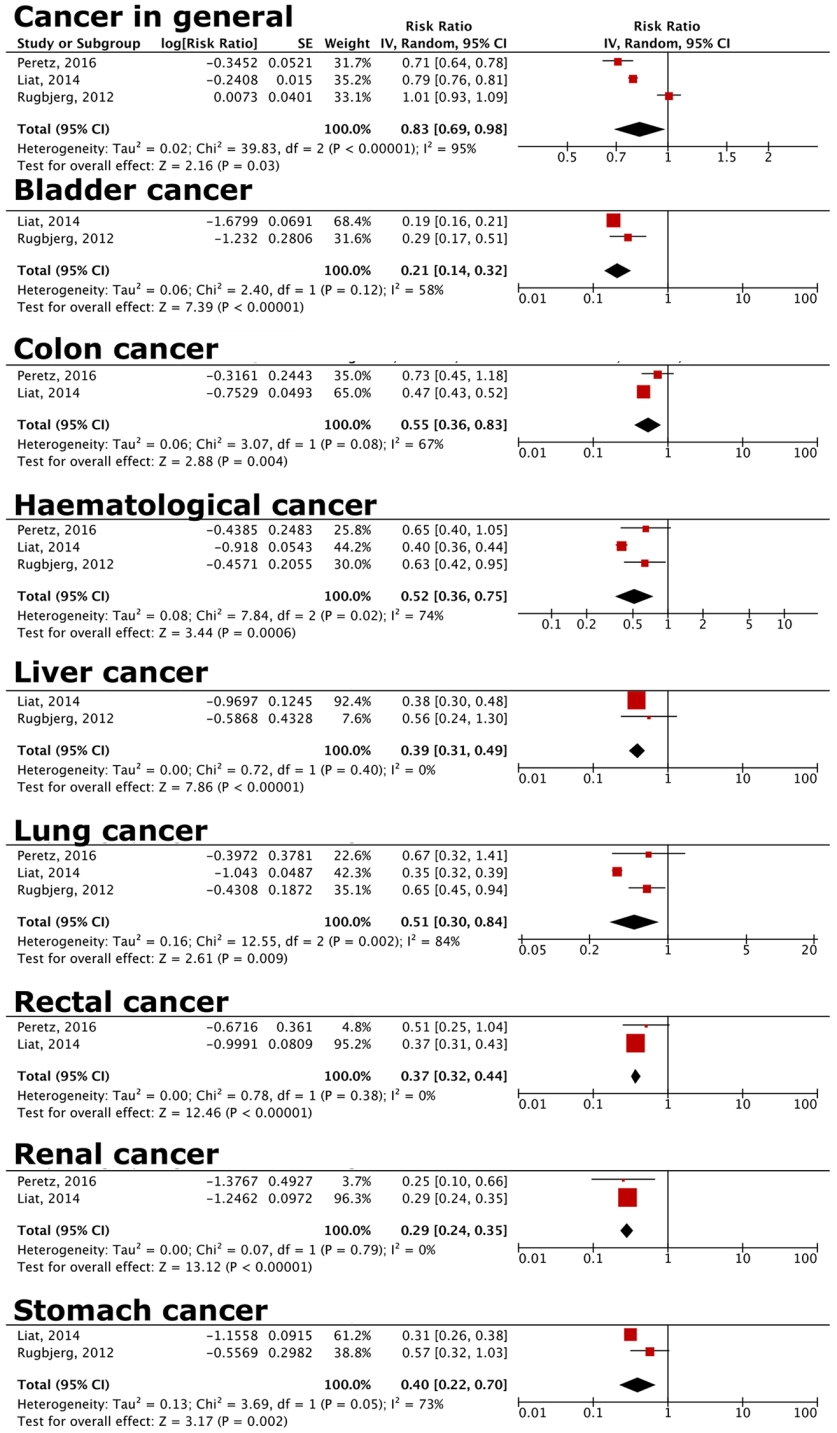
**Forest plot comparing risks of cancer in general and specific cancers for female vs. male PD patients.** Female PD patients have decreased risks of overall cancer, and bladder, colon, haematological, liver, lung, and rectal cancer compared to male PD patients. Details of specific cancers included in each cancer group are listed in Supplementary Table 6A–6C.

Female PD patients have a decreased general cancer risk compared to male PD patients in this analysis (RR = 0.83, 95% CI = 0.69–0.98; Figure 4). In terms of specific cancers, there was a decreased risk of bladder (RR = 0.21, 95% CI = 0.14–0.32), colon (RR = 0.55, 95% CI = 0.36–0.83), hematological (RR = 0.52, 95% CI = 0.36–0.75), kidney (RR = 0.29, 95% CI = 0.24–0.35), liver (RR = 0.39, 95% CI = 0.31–0.49), lung (RR = 0.51, 95% CI = 0.30–0.84), rectal (RR = 0.37, 95% CI = 0.32–0.44), and stomach cancer (RR = 0.40, 95% CI = 0.22–0.70; Figure 4).

## DISCUSSION

We showed that PD patients have decreased subsequent cancer risks (RR = 0.87, 95% CI = 0.81–0.93), with a reduced risk of colon, rectal, and colorectal cancer (RR = 0.77, 95% CI = 0.63–0.94), lung cancer (RR = 0.62, 95% CI = 0.48–0.80). There was an increased brain cancer (R = 1.48, 95% CI = 1.02–2.13) and melanoma risk (R = 1.76, 95% CI = 1.23–2.50). Compared to idiopathic PD, LRRK2-G2019S carrier patients had an increased risk of cancer in general (RR = 1.26, 95% CI = 1.09–1.46), especially for brain (RR = 2.41, 95% CI = 1.06–5.50), breast (RR = 2.57, 95% CI = 1.19–5.58), colon (RR = 1.83, 95% CI = 1.13–2.99), and hematological cancers (RR = 2.05, 95% CI = 1.07–3.92). Female PD patients have a decreased general cancer risk compared to male PD patients in this analysis (RR = 0.83, 95% CI = 0.69–0.98).

Several hypotheses could explain the above observations. These include opposing molecular pathways between PD and cancer, lifestyle changes in PD patients following PD diagnosis, and an increased rate of healthcare utilization and surveillance among PD patients compared to non-PD individuals.

### Opposing molecular pathways of PD and cancer

PD involves degeneration of the dopamine producing cells of the substantia nigra, while cancer, with its proliferative nature [23], lies on the opposite end of the spectrum. Several PD-related genes have been found to possibly mediate the relationship between PD and subsequent cancer. These genes include LRRK2, PARK2, a tumor suppressor gene, PARK5, coding for the ubiquitin carboxyl-terminal hydrolase-L1 (UCH-L1) enzyme involved in ubiquitin-recycling, PARK7 (DJ-1), a strong anti-oxidant, and PARK6 (PINK1), a cell death and cell cycle regulator [23]. Oxidative damage, alterations in protein ubiquitination, and cell cycle dysregulation have been implicated in cancer pathogenesis [24]. Therefore, the PARK family proteins involved both in PD and regulation of replication stress can possibly mediate both pathologies.

### Lifestyle changes in PD patients

Amongst PD patients, a ‘Parkinsonian personality’ characterized by low novelty seeking (NS) and high harm avoidance (HA) behavior, possible resulting from decreased dopaminergic stimulation, has been described [25]. NS behaviors include impulsivity, reward seeking, and exploration of novel experiences, while HA behaviors include pessimism, worry, and avoidance due to uncertainty [25]. While epidemiological research in this domain is lacking, PD patients may possibly be engaging in less risky lifestyle behaviors like smoking and adopting healthier habits of increased physical activity and eating balanced diets. These lifestyle attributes are related to a decreased cancer risk [26], contributing to lower cancer risks seen in our study.

### Increased healthcare utilization and surveillance in PD patients

Tremors, rigidity, and bradykinesia significantly reduces one’s ability to perform daily activities [27]. PD patients also tend to have more comorbidities, including obesity, diabetes mellitus, and cardiac pathologies [27].

Furthermore, PD treatment with dopaminergic agonists may lead to complications such as cardiac fibrosis and arrhythmias [27]. The combination of PD-related symptoms, multiple comorbidities, on top of PD treatment effects has necessitated increased expenditure and healthcare utilization rates among PD patients in countries like Brazil [28] and the United States [29]. In the United States, states with higher PD prevalence have increased awareness and recognition of PD symptoms, further driving healthcare seeking behaviours [29] and comprehensive medical care involving not only neurologists, but also internal medicine physicians [27].

This possibly explains, at least partly, the decreased general cancer risk amongst PD patients, through earlier detection and management.

### Risks of specific cancers following PD diagnosis

#### Smoking-related cancers

Evidence of an inverse relationship between smoking and PD development is abundant, with this negative correlation intensified by smoking duration and dose [30]. Chemical substances in cigarettes and cigarette smoke, including nicotine and 2,3,6-trimethyl-1,4-napthoquinone (TMN), have been proposed to influence this inverse relationship [30]. TMN is a MAO inhibitor that reduces neurodegeneration induced by metabolites like MPTP [21, 30], while nicotine, acting on the striatal nicotinic receptors, can augment dopamine release [30], or have protective effects leading to dopaminergic neuron survival [21].

Furthermore, the decreased NS and increased HA behaviors previously detailed may lead to decreased smoking rates in PD patients. The biochemical effects of smoking on PD development, coupled with decreased smoking habits, may result in an overall reduced smoking-related cancer observed.

#### Brain cancers

Emerging research has pointed towards the neuroprotective effects of the gut microbiome through reducing proinflammatory cytokine production, inducing secretion of the anti-inflammatory interleukin IL-10, and promoting development of Treg cells that play a role in immunosuppression [31]. These effects are due to metabolites produced by the microbiota, especially short chain fatty acids (SCFAs) that have anti-inflammatory, neuroprotective, and anti-oxidant effects [32]. It is also postulated that these metabolites and the mediators induced as a result, could affect blood-brain barrier integrity, influencing susceptibility to neural insults [31].

Gut microbiome biodiversity alteration in PD patients, specifically in the abundance of bacteria in the phylae *Firmicutes, Bacteroidetes*, and *Proteobacteria* [33], have been reported. Decreased microbial biodiversity likely led to decreased SCFA production and therefore neuroprotective effects, possibly explaining the increased brain cancer risks.

### Melanoma

Melanoma has consistently been reported to be more prevalent among PD patients. Several lines of evidence, from shared risk factors, common biochemical pathways, and genes, have been put forth to explain this relationship.

Ye et al. (2020) [34] outlined several overlapping characteristics between PD and melanoma: In terms of ethnicity, PD and melanoma were more common in Whites, with both rates increased in individuals with fair skin tones and red hair. In terms of lifestyle behaviors, decreased smoking rates in PD patients were correlated with increased melanoma risks, while coffee consumption was associated with both a decreased PD and melanoma risk.

Biochemically, the pigmentation pathway is shared for melanin production in the skin and neuromelanin in the brain from tyrosine [11]. Melanin in the skin protects cells against DNA damage induced by UV radiation, while neuromelanin is a crucial neuroprotective pigment in the dopaminergic neurons by sequestering reactive oxygen species and metal ions [11]. Alterations in this common pathway resulting in decreased melanin and neuromelanin production may therefore make skin cells more susceptible to genetic instability, and dopaminergic neurons more vulnerable to oxidative damage, possibly linking PD and melanoma.

Loss of heterozygosity of PARK2, LRRK2 mutations causing neuronal cell death and neurotoxicity, BRAF kinase alterations, and PARK7 oncogene activation with subsequent melanoma development are possible underpinning genetic pathophysiologiy [11, 34].

The combined effect of common risk factors, shared biochemical pathways, and overlapping genes provide strong evidence linking the positive correlation between PD and melanoma occurrence.

### Cancer risks in LRRK2-G2019S PD carriers

LRRK2, a protein kinase gene, is most commonly implicated in familial PD [35]. LRRK2 promotes aggregation of α-synuclein into Lewy bodies and tau tangles [35]. In addition, these mutations also contribute to neurodegeneration in PD by driving cells towards a pro-inflammatory state, increasing oxidative stress, and disrupting mitochondrial functions and the autophagy-lysosomal system [35].

Inflammation, oxidative damage, mitochondrial dysfunction and disruption of the autophagy-lysosomal system are processes unique not only to PD development, but also cancer [36]. It is therefore unsurprising to find increased cancer risks amongst LRRK2-G2019S PD patients in our study, with LRRK2 promoting PD-associated neurodegeneration and cancer-related pathogenesis pathways. Interestingly, in addition to the expression of various LRRK2 mutations, namely R1441C, R1441G, R1441H, and G2019S in the brain [37], LRRK2 has been found in peripheral blood cells [38], gut [39], and in the lung and breast [40]. These expression patterns mirror our findings of increased brain, breast, colon, and hematological cancers, further strengthening the association between LRRK2 and cancer and increasing the value of targeting LRRK2 for therapeutic treatment of both PD and cancer.

LRRK2 has been identified to be a candidate prognostic biomarker for clear cell renal cell carcinoma [41]. Yang et al. [41] showed that there was up regulation (confirmed on immunohistochemical and protein studies) of LRRK2 expression that was associated with DNA methylation in this cancer. Interestingly, somatic LRRK2 truncating or deletion mutations have been identified in malignant mesothelioma and LRRK2 expression was absent or downregulated in primary tumor cell lines [42]. How this tumor suppressor change predispose to cancers still needs to be investigated. A specific LRRK2 rs10878441 CC genotype has been linked to a poorer prognosis in Chinese breast cancer patients [43]. High LRRK2 expression has also been associated with poorer survival in ovarian cancer [44]. It was also demonstrated that inhibiting LRRK2 promoted toxicity of PARP inhibitor by reducing homologous recombination-mediated DNA double strand break repair [44]. LRRK2 is also involved in the ATM-Mdm2-p53 pathway that regulates cell proliferation in response to DNA damage [45]. These clinical and experimental observations provide support linking LRRK2 to cancer.

### Decreased cancer risk in female PD patients

The protective role of estrogen has been well documented in dopaminergic neurons [46], adipose tissues, skeletal muscles, macrophages, and immune cells [47]. Estrogen is neuroprotective, reducing the oxidative damage from dopamine, iron, and calcium [46] that contribute to PD development. In other non-neuronal cells, estrogen modulates fuel metabolism, specifically of lipids, amino acids, and glucose [47], which are commonly dysregulated in cancer cells [36]. This can possibly explain why females with higher lifetime estrogen exposures, and women who have used estrogen therapy have decreased PD [46], as well as cancer risks found in our analysis.

LRRK2 and estrogen have opposing effects on similar domains of inflammation, oxidative stress, and metabolism, with the former toxic and the latter protective in neurons and non-neuronal cells. It may therefore be reasonable to postulate that LRRK2 mutation effects may override the protection afforded by estrogen in female carriers, resulting in more severe PD symptoms and increased cancer risks.

### Implications of study

The identification of potential healthier lifestyle choices and more frequent healthcare monitoring provides increased impetus to encourage PD patients to adopt lifestyle changes and follow-up adherence to reduce both PD progression and cancer development. Furthermore, the involvement of PARK family genes in PD and cancer pathogenesis opens a new therapeutic angle through targeted downregulation of these genes to reduce risk of contracting either or both pathologies.

While general cancer risk in PD patients was decreased, increased risks of brain cancers and melanoma were found. This prompts a need for more frequent screening for early signs and symptoms of these neoplasms. The gut-brain microbiome’s effects and decreased gut biodiversity in PD patients suggest that diet regulation and probiotics to promote improved gut health may be a preventive measure against brain cancers. The common pathway involved in melanin and neuromelanin production, implicated in both melanoma and PD, indicates a possible treatment strategy focused on altering the enzyme kinetics as a means of reducing melanoma risks.

The opposing effects of LRRK2 and estrogen on PD and cancer development highlight the potential utility of estrogen replacement to slow PD progression and severity, as well as cancer development in PD patients. This is supported by a prior study [48] indicating that estrogen has beneficial effects on neurons in the nigrostriatum. While further analysis is required to determine the contribution of gender and estrogen effects on the increased cancer risks in LRRK2-PD patients, our findings suggest the potential utility of hormonal therapy as a dual preventive measure for PD and cancer.

### Comparison to previous meta-analysis

Two other meta-analyses investigating the relationship between PD and subsequent cancer development were conducted in 2010 [49], 2019 [50] respectively. However, our study has several strengths.

First, we uniformly extracted unadjusted RRs from the included studies, or manually calculated it from the data provided in the papers or by the authors. In contrast, Bajaj et al. (2010) [49] and Zhang and Liu (2019) [50] extracted and treated adjusted and unadjusted ORs, RRs, SIRs, and HRs equally, under the assumption that PD and cancer are rare conditions. However, with increased prevalence of both diseases [51, 52], it is incorrect to make the assumption, and hence only RRs would reflect the true risk of developing cancer after PD. Additionally, since different studies adjusted their results based on different factors, the actual PD effect on subsequent cancer risk can only be compared equally using unadjusted RRs, as in this case.

Second, we provided a concise account of cancer risks in specific PD populations, comparing between male and female patients and between LRRK2-PD and idiopathic PD patients. Although the negative association between PD and subsequent cancer development found in this study was similar to that of previous meta-analysis [49, 50] additional analyses conducted showed that the decreased risks were more significant in female and idiopathic PD patients.

Identification of specific at-risk subgroups can facilitate management strategies encompassing increased screening and surveillance, lifestyle changes, and hormonal replacement as promising therapeutic options. This study therefore provides a holistic review of not just the relationship shared between PD and cancer, but the multiple factors and probable treatment options for PD patients.

### Study limitations

First, the effects of comorbidities and level of tobacco use could not be analyzed as the information were unavailable. Second, as the included studies were mostly conducted in Western populations, the generalizability of the current findings to an Asian population is unclear.

In conclusion, we demonstrated that PD patients have a reduced risk of colon, rectal, colorectal cancer and lung cancers and an increased risk of brain cancer and melanoma. LRRK2-G2019S carriers have an increased cancer risk, in particular for brain, breast, colon and blood cancers and female gender was associated with a reduced risk of bladder colon, hematological, kidney, liver, lung, rectal, and stomach cancer.

Future gene-environmental and lifestyle prospective studies will be able to identify factors that may modulate the association between PD and cancer. Functional studies in experimental models to elucidate the pathophysiology of PD and cancer contributed by kinase functions and targets of LRRK2 in the cell cycle may facilitate identification of therapeutic targets.

## METHODS

### Search strategy

Database search was conducted on PubMed, Web of Science, and SCOPUS to identify published articles between 1 January 2010-30 August 2020 investigating the incidence and prevalence of cancer following PD diagnosis. “Parkinson disease”, “Neoplasm”, “Cancer”, and “Epidemiological studies” were entered as search topics or medical subject headings and connected with Boolean operators. Where applicable, filters were applied to limit studies to those conducted in humans, in English, and were in full text. The search strategy is detailed in Supplementary Methods.

Searches were performed for each database and were updated until 1 June 2021.Titles and abstracts were screened independently by two reviewers (J.Y.S.L and J.H.N) against a set of pre-defined eligibility criteria. Potentially eligible studies were selected for full-text analysis. Additional relevant studies were identified by manually examining the references provided in the published studies identified initially during the database search.

### Eligibility criteria

Studies eligible for inclusion in the primary analysis investigated the impact of PD on subsequent cancer development. Data were reviewed to ensure that subjects recruited in the studies were cancer-free before PD diagnosis, regardless of subsequent cancer development. This was done through appraising the study cohorts employed between two independent reviewers. Studies investigating cancer’s effect on subsequent PD development, and on cancer risk in relatives of PD patients were not considered. Resolution of disagreements were by consensus after discussion.

Of the studies identified through the initial database search, eight investigated the effect of pharmaceutical PD treatment on cancer risk [17, 53–59], five looked at the impact of genetic variants, such as LRRK2 on cancer development [13, 16–18, 60] and six investigated PD’s effect on the development of specific cancers [3, 22, 56, 58, 61, 62]. Another study [63] investigated PD’s impact on subsequent cancer development but did not report the outcome statistics, and was excluded from primary analysis. While these studies were not included in the primary analysis, they were included in subgroup analyses to determine the effect of gender, PD treatment, PD-related genetic variants, or PD’s effect on specific cancers.

Studies meeting the eligibility criteria were then analyzed in detail to ensure that there were no overlapping study cohorts. Four Taiwanese [6, 9, 64, 65] and two Israeli studies [19, 66] utilized the same study cohort in their respective countries. The study that employed the most study subjects and tracked the development of the most cancers in each of the two countries was ultimately chosen.

### Exclusion criteria

This analysis excluded papers that were non-English and conducted in non-human subjects. Non-original research papers, laboratory-based, and epidemiological studies with no clinical characteristics reported were also not considered. Case series and case reports were excluded according to recommendations by the Cochrane Statistical Methods Group and in accordance with methodologies of previously published meta-analyses [67].

### Data extraction

Information from the studies were extracted by two independent reviewers (J.Y.S.L and J.H.N). These included the number of subjects recruited, demographic details inclusive of the mean age, gender distribution, and country where the study was conducted. Information pertaining to the study included the study design, the adjustment applied to the outcome variables (risk ratio; RR, hazard ratio; HR, odds ratio; OR, and standardised incidence rate; SIR).

### Outcome

Overall and specific cancer risk analyzed by each study and the number of subjects who developed each kind of cancer were extracted. We used the unadjusted RR as the common outcome measurement for comparison between all studies. If only adjusted RRs or adjusted or unadjusted ORs were reported, unadjusted RR values were manually calculated.

Unadjusted HRs and SIRs were considered interchangeable with the unadjusted RR [68]. If studies reported only adjusted HRs, effort was invested to contact the authors to obtain the unadjusted values. For conversion of adjusted RRs and adjusted ORs to unadjusted RRs, and for ACR computation, we contacted the authors of the studies to determine if the number of cases of subsequent cancer in PD patients and control subjects were reported.

Of the fourteen papers, three provided adjusted HRs [64, 69, 70], two provided unadjusted SIRs [4, 19], one provided unadjusted ORs [15], three provided adjusted RRs [20, 71, 72], while five provided adjusted ORs [14, 73–76]. Of the studies reporting adjusted RRs and adjusted ORs, only one author [14] was able to supplement with unadjusted ORs. Other authors were unable to assist in our analysis due to a lack of access or unavailability of study data.

### Assessment of study quality

The risk of bias (RoB) analysis was conducted using the Newcastle-Ottawa Scale (NOS). NOS scores were subsequently converted to Agency for Healthcare Research and Quality (AHRQ) ratings to classify the studies as of ‘Good’, ‘Fair’ or ‘Poor’ quality. The RoB and AHQR framework used for study assessment in this meta-analysis are detailed in Supplementary Methods. Two reviewers (J.Y.S.L and J.H.N) assessed the quality of all included studies and discussed discrepancies until consensus was reached. The risk of bias analysis for cohort and case-control studies are detailed in Supplementary Table 5A and 5B respectively.

### Subgroup analyses

Besides analyzing the relationship between PD and the risk of subsequent cancer in general, and that of specific cancers through the primary and subgroup analysis, secondary analyses were conducted. These subgroup analyses investigated the effect of genetic variants implicated in PD development, PD treatment, and gender on subsequent cancer development. Studies included in these subgroup analyses were identified during the database search but did not meet the inclusion criteria for primary analysis. We conducted subgroup analyses using these studies since both cancer and PD are multifactorial. Similar variables were extracted for the secondary analyses as for the primary analyses. Details and results of the studies included are provided in the subsequent sections.

### Statistical analysis

Review Manager (Review Manager (RevMan) [Computer program]. Version 5.4. The Cochrane Collaboration, 2020) was used for data analysis in the present study. Type I error was fixed at 5% and 95% confidence intervals were reported for all calculations.

#### Heterogeneity between studies

Heterogeneity between the studies was evaluated using the *Q* test and I^2^. *P* values for the I^2^ statistics were computed by chi-square distribution of Cochran *Q* test. Random effect models were used to pool the results and to allow for differences in the treatment effect from study to study (sampling variability across studies). Subgroup analyses on PD’s effect on subsequent development of specific cancers were conducted to assess the heterogeneity source.

#### Sensitivity analysis

Sensitivity analysis was conducted to assess the robustness of the present study. The meta-analysis was conducted twice, once with all studies included, and once after excluding studies rated ‘Poor’ by AHRQ standards for sensitivity analysis.

#### Publication bias

Publication bias of the included studies was assessed using the funnel plot (Supplementary Figure 1A–1C).

## Supplementary Materials

Supplementary Methods

Supplementary Figure 1

Supplementary Tables
