# Supplementary material for: Parkinson’s disease and cancer: a systematic review and meta-analysis on the influence of lifestyle habits, genetic variants, and gender
Source: Aging (Albany NY). 2022 Mar 5;14(5):2148–73. doi: 10.18632/aging.203932 (PMC8954974; doi:10.18632/aging.203932)
Supplement: Supplementary Methods [file aging-14-203932-s001.pdf]

SUPPLEMENTARY METHODS

Database search strategy

Pubmed search – 27 August 2020

- Search chain: (((neoplasm[MeSH Terms]) OR (cancer) AND ((fft[Filter]) AND (humans[Filter]))) AND ((parkinson's) OR (parkinson's disease[MeSH Terms]) AND ((fft[Filter]) AND (humans[Filter])))) AND ((((epidemiological studies[MeSH Terms]) OR (cohort studies[MeSH Terms])) OR (case control studies[MeSH Terms])) OR (observational studies) AND ((fft[Filter]) AND (humans[Filter])))
- Date range: 2010–2020
- Number of identified results: 322
- Number of shortlisted results: 47

SCOPUS search – 28 August 2020

- Search chain: (**'parkinson disease'**/mj OR **'parkinson disease'** OR **'parkinson`s disease'** OR **'parkinsons disease'** OR **'paralysis agitans'** OR **'parkinson dementia complex'** OR **'parkinson disease, postencephalitic'** OR **'parkinson disease, secondary'** OR **'parkinson disease, symptomatic'**) AND **'neoplasm'**/mj AND (**'incidence'**/exp OR **'incidence'** OR **'incidence rate'** OR **'rate, incidence'** OR **'prevalence'**/exp)
- Date range: 2010–2020

Web of Science search – 30 August 2020

- Search chain:

  Step 1: Cancer (Topic)

  Step 2: Parkinson's disease (Topic)

  Step 3: Epidemiological study (Topic)
- Search: 1 AND 2 AND 3
- Filters: English
- Date range: 2010–2020

Risk of bias (RoB) analysis using the Newcastle Ottawa Scale

The Newcastle Ottawa Scale (NOS) assesses study quality using a 9-point scale broadly divided into the following three categories:

a. Study selection – 4 points

b. Comparability – 2 points

c. Exposure – 3 points.

The detailed criteria for RoB analysis using the NOS scale for case-control and cohort studies are detailed elsewhere.

Subsequent conversion of NOS scores to Agency for Healthcare Research and Quality (AHRQ) standards was done classify the studies as being 'Good', 'Fair' or 'Poor' quality, based on the following criteria:

a. Good – 3 or 4 stars in selection domain AND 1 or 2 stars in comparability domain AND 2 or 3 stars in outcome/exposure domain.

b. Fair – 2 stars in selection domain AND 1 or 2 stars in comparability domain AND 2 or 3 stars in outcome/exposure domain.

c. Poor – 0 or 1 star in selection domain OR 0 stars in comparability domain OR 0 or 1 stars in outcome/exposure domain.
