# Supplementary material for: Parkinson’s disease and cancer: a systematic review and meta-analysis on the influence of lifestyle habits, genetic variants, and gender
Source: Aging (Albany NY). 2022 Mar 5;14(5):2148–73. doi: 10.18632/aging.203932 (PMC8954974; doi:10.18632/aging.203932)
Supplement: Supplementary Figure 1 [file aging-14-203932-s002.pdf]

SUPPLEMENTARY FIGURE

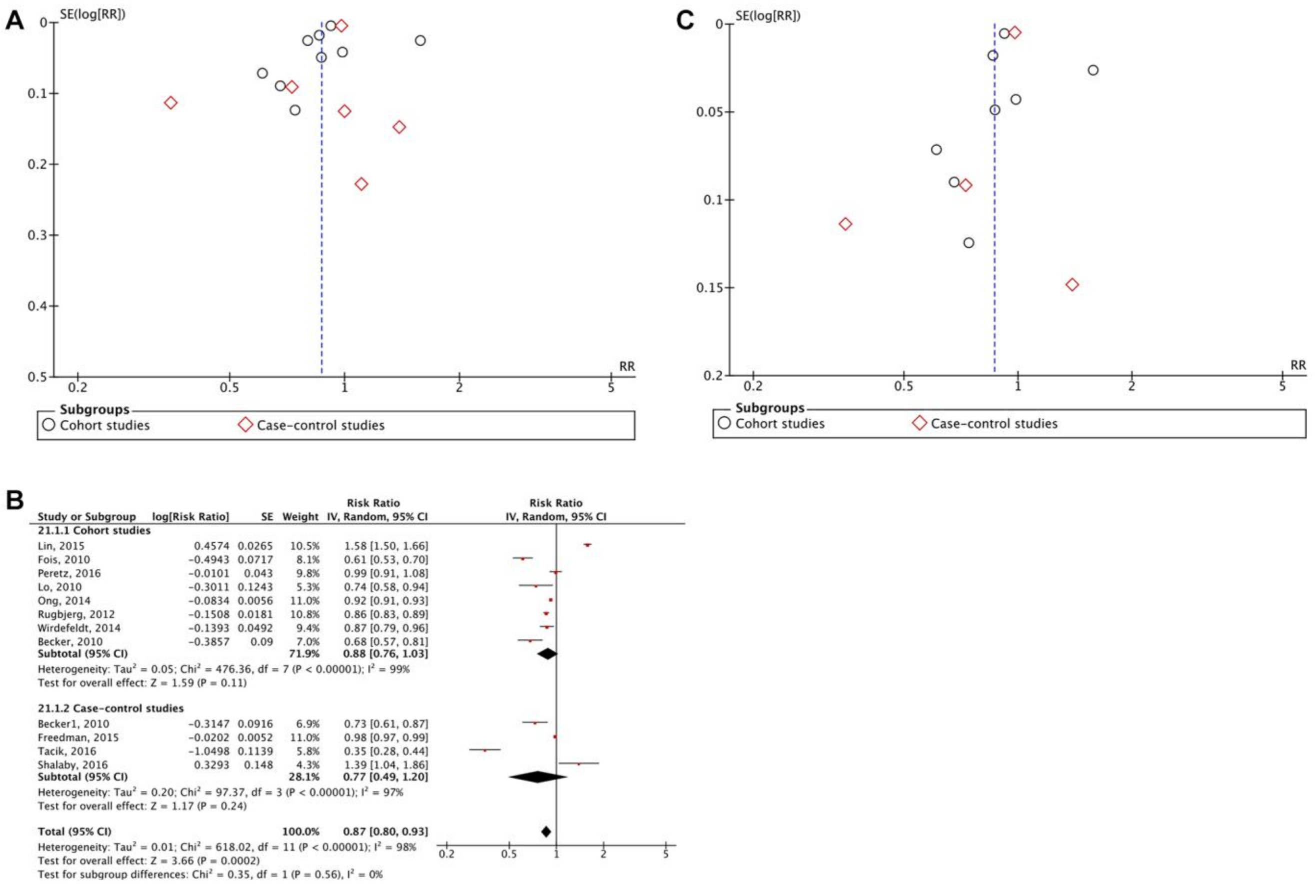

Supplementary Figure 1. (**A**) Funnel plot for assessment of publication bias in the included studies before sensitivity analysis. (**B**) Forest plot of the association between PD and relative risk of cancer in general, after sensitivity analysis. (**C**) Funnel plot of included studies after sensitivity analysis.
