# Supplementary material for: Parkinson’s disease and cancer: a systematic review and meta-analysis on the influence of lifestyle habits, genetic variants, and gender
Source: Aging (Albany NY). 2022 Mar 5;14(5):2148–73. doi: 10.18632/aging.203932 (PMC8954974; doi:10.18632/aging.203932)
Supplement: Supplementary Tables [file aging-14-203932-s003.pdf]

SUPPLEMENTARY TABLES

Supplementary Table 1. QUOROM Statement checklist.

Heading	Subheading	Descriptor	Reported? (Y/N)	Page Number
**Title**		Identify the report as a systematic review	Y	1
**Abstract**		Use a structured format	Y	2
	Objectives	The clinical question explicitly	Y	2
	Data sources	The databases (i.e., list) and other information sources	Y	2
	Review methods	The selection criteria (i.e., population, intervention, outcome, and study design); methods for validity assessment, data abstraction, and study characteristics, and quantitative data synthesis in sufficient detail to permit replication	Y	2
	Results	Characteristics of the RCTs included and excluded; qualitative and quantitative findings (i.e., point estimates and confidence intervals); and subgroup analyses	Y	2
	Conclusion	The main results	Y	2
		**Describe**		
**Introduction**		The explicit clinical problem, biological rationale for the intervention, and rationale for review	Y	3–4
**Methods**	Searching	The information sources, in detail (e.g., databases, registers, personal files, expert informants, agencies, hand-searching), and any restrictions (years considered, publication status, language of publication)	Y	17
	Selection	The inclusion and exclusion criteria (defining population, intervention, principal outcomes, and study design	Y	17–18
	Validity assessment	The criteria and process used (e.g., masked conditions, quality assessment, and their findings)	Y	17–18
	Data abstraction	The process or processes used (e.g., completed independently, in duplicate)	Y	17
	Study characteristics	The type of study design, participants' characteristics, details of intervention, outcome definitions, and how clinical heterogeneity was assessed	Y	19
	Quantitative data synthesis	The principal measures of effect (e.g., relative risk), method of combining results (statistical testing and confidence intervals), handling of missing data; how statistical heterogeneity was assessed; a rationale for any a-priori sensitivity and subgroup analyses; and any assessment of publication bias	Y	20–21
**Results**	Trial flow	Provide a meta-analysis profile summarising trial flow (see figure)	Y	7–10
	Study characteristics	Present descriptive data for each trial (e.g., age, sample size, intervention, dose, duration, follow-up period)	Y	Refer to Table 1
	Quantitative data synthesis	Report agreement on the selection and validity assessment; present simple summary results (for each treatment group in each trial, for each primary outcome); present data needed to calculate effect sizes and confidence intervals in intention-to-treat analyses (e.g., 2X2 tables of counts, means and SDs, proportions)	Y	Refer to Table 1
**Discussion**		Summarise key findings; discuss clinical inferences based on internal	Y	7–16

and external validity; interpret the results in light of the totality of available evidence; describe potential biases in the review process (e.g., publication bias); and suggest a future research agenda

Supplementary Table 2. Papers excluded from primary analysis and the reasons for exclusion.

First author	Reasons for rejection
Sun, 2011	Overlapping database and time period, smaller group of patients and controls, fewer number of cancers studied compared to Lin (2015)
Tang, 2016	Overlapping database and time period, fewer number of cancers studied compared to Lin (2015)
Liao, 2015	Overlapping database and time period, fewer number of cancers studied compared to Lin (2015)
Lerman, 2018	Overlapping database and time period, fewer number of cancers studied compared to Peretz (2016)
Inzelberg, 2011	Studied impact of PD on melanoma instead of cancer in general
Bertoni, 2010	Studied impact of PD on melanoma instead of cancer in general
Boursi, 2016	Studied impact of PD on colorectal cancer instead of cancer in general
Ryu, 2020	Studied impact of PD on skin cancer instead of cancer in general
Jespersen, 2016	Studied impact of PD on prostate cancer instead of cancer in general
Constantinescu, 2013	Studied impact of PD on melanoma instead of cancer in general

Supplementary Table 3. Characteristics of all studies included in the comparison between LRRK2-PD and idiopathic PD patients.

No.	Author	Study design	Country	Sample size	Females (%)	Mean age (SD)	Adjustment	Cancer (s) reported
1	Saunders-Pullman, 2010	Cohort	USA	31 LRRK2-PD patients<br>132 iPD patients	75 (46.0%)	70.2 (median)	Smoking, gender	Non-skin, renal, breast, lung, prostate, haematological, reproductive
2	Agalliu, 2019	Case-control	Europe, Israel, USA	257 LRRK2-PD patients<br>712 iPD patients<br>218 non-PD controls	553 (46.9%)	67.3 (10.81)	Age, sex, Ashkenazi Jews ethnicity (fixed effect) and study centre (random effect), smoking status, BMI	Cancer in general, skin, melanoma, head and neck, lung, esophageal, colon, liver, pancreatic, thyroid, kidney, bladder, brain, leukemia, lymphoma, hormone-related, breast, ovarian, endometrial, cervical, prostate, testicular
3	Warø, 2018	Case-control	Norway	103 LRRK2-PD patients<br>830 iPD patients	361 (38.7%)	71.2 (11.6)	Age, sex	Colorectal, lung, breast, prostate, kidney, bladder, thyroid, lymphoma/haematologic, meningioma, non-skin, others unspecified
4	Ruiz-Martínez, 2014	Case-control	Spain	95 LRRK2-PD patients<br>637 iPD patients<br>176 non-PD controls	448 (49.3)	71.2 (11.8)	NR	Cancer in general, melanoma, lung, bladder, colon, kidney, breast, ovarian, prostate, hormonal, haematologic, meningioma, others unspecified
5	Inzelberg, 2012	Case-control	Israel	79 LRRK2-PD patients<br>411 iPD patients	191 (39.0%)	69.8 (11.1)	Age	Cancer in general, lung, breast, prostate, colon, stomach, haematologic, reproductive, renal, skin, melanoma, non-melanoma skin, others unspecified
6	Agalliu, 2015	Case-control	Israel, Norway, Spain, USA	177 LRRK2-PD patients<br>1372 iPD patients	680 (43.9)	70.9 (10.8)	Adjustment (1): Age at time of the first cancer diagnosis, or age at the last clinic visit<br><br>Adjustment (2): Age as fixed effect, study centre as random effect<br><br>Adjustment (3): Age and ethnicity (Ashkenazi Jewish vs. others) as fixed effects and study centre as random effects	Cancer in general, skin, melanoma, non-skin, lung, bladder, breast, ovarian, prostate, colon, kidney/renal, haematologic/lymphoma, meningioma

Abbreviations: LRRK2: Leucine-rich repeat kinase 2; iPD: Idiopathic PD; NR: Not reported.

Supplementary Table 4. Characteristics of all studies included in the comparison between female and male patients.

No.	Author	Study design	Country	Sample size of PD patients	Mean age (SD)	Adjustment	Cancer (s) reported
1	Sun, 2011	Cohort	Taiwan	2395 females (48.3%)<br>2562 males (51.7%)	63.5 (20.5)	Model 1: Unadjusted<br>Model 2: Adjusted for age, sex, occupation<br>Model 3: Adjusted for age, sex, occupation, HTN, DM, hyperlipidemia, heart disease	Cancer in general
2	Peretz, 2016	Cohort	Israel	3297 females (46.3%)<br>3828 males (53.7%)	71.3 (10.6)	Age, chronological year, sex	Cancer in general, breast, colon, CNS, kidney, leukemia, lung. Lymphoma, melanoma, ovary, pancreas, prostate, rectum, thyroid
3	Liat, 2014	Cohort	UK	94254 females (43%)<br>124940 males (57%)	NR	NR	Cancer in general, bladder, bone, brain, breast, cervix, colon, upper GI, kidney, larynx, lymphoid leukemia, myeloid leukemia, liver, lung, Hodgkin's lymphoma, non-Hodgkin's lymphoma, malignant melanoma, multiple myeloma, nasopharynx, meninges, oesophageal, ovarian, pancreatic, prostate, rectum, salivary gland, non-melanoma skin cancer, stomach, testis, thyroid, uterine body
4	Rugbjerg, 2012	Cohort	Denmark	9631 females (47%)<br>10712 males (53%)	NR	NR	Cancer in general, malignant melanoma, nonmelanoma skin cancer, buccal cavity, stomach, colorectal, liver, lung, urinary bladder, myeloid leukemia, gallbladder/biliary tract, brain, non-Hodgkin lymphoma, multiple myeloma, lymphatic leukemia

Abbreviations: HTN: Hypertension; DM: Diabetes mellitus; NR: Not reported.

Supplementary Table 5A. Risk of bias analysis for cohort studies.

Author	Selection				Comparability	Exposure			Total quality score	Quality rank
	Representativeness of exposed cohort	Selection of the non-exposed cohort	Ascertainment of exposure	Demonstration that outcome of interest was not present at start of study	Comparability of cohorts on the basis of the design or analysis	Assessment of outcome	Was follow-up long enough for outcomes to occur	Adequacy of follow up of cohort		
Lin, 2015	*	*	*	*	**(age, sex, index year)	*(National Cancer Registry Database + follow-up)	*(Until diagnosis of malignant disease, death, lost to follow-up, withdrew from database, until end of study date)		8	Good
Peretz, 2016	*	*	*		*(age, sex)	*(MHS cancer registry)	*(Until death, leaving HMO, study closure, ~10y to reflect onset of motor symptoms and diagnosis)		6	Good
Park, 2019	*	*	*	*	**(age and sex)		*(Followed up for cancer development until 2016, from 2010)		7	Poor
Liat, 2014	*	*	*	*	**(age, sex, calendar year of 1st recorded admission, region of residence, quintile of patients' Index of Deprivation score)	*(Search for malignant cancer using ICD records and individual cancer outcomes)	*(1 Jan 1999–31 Dec 2011)		8	Good
Rugbjerg, 2012	*	*	*	*	**(age, sex, calendar period)	*(Danish Cancer Registry)	*(From 1/1/1977–2008)		7	Good
Wirdefeldt, 2014	*	*	*		**(birth year, sex)	*(Swedish Cancer Register)	*(1958–2009)		7	Good
Becker, 2010[1]	*	*	*	*	**(Age, gender, general practice, diagnosis date, years of history in the GPRD prior to diagnosis date)	*	*(accumulated person-time until patient developed an incident cancer diagnosis, died, the medical record ended, or end of study was reached – 31 December 2005)		8	Good
Fois, 2010	*	*	*		*(age at entry, sex, calendar year of 1st recorded admission, interval from study entry and district of residence)	*(ORLS database)	*(date of subsequent admission for cancer, death, or 31 March 1999)		7	Good
Lo, 2010	*	*	*		*(birth year, gender, respondent type)	*KPNCCR database	*(from time of 1st membership to 29 February 2008)		7	Good

[1]Both case-control and cohort studies were conducted.

Supplementary Table 5B. Risk of bias analysis for case-control studies.

Author	Selection				Comparability	Exposure			Total quality score	Quality rank
	Is the case definition adequate?	Representativeness of the cases	Selection of controls	Definition of controls	Comparability of cases and controls	Ascertainment of exposure	Same method of ascertainment for cases and controls	Non-response rate		
Freedman, 2015	*	*	*	*	**(age and sex)	*(ICD codes used)	*		8	Good
Tacik, 2016	*	*		*	**(age and sex)	*(UKPDSBB, Mayo Clinic specialists)	*		7	Good
Shalaby, 2016	*	*	*	*	**(age and sex)	*(CUMC, published diagnostic criteria)	*		8	Good
Becker, 2010[1]	*	*	*	*	**(age, gender, calendar time)	*(records and codes)	*		8	Good
Agalliu, 2019	*	*	*		**(age, ethnicity)	(self-reported questionnaire)	*		6	Poor
Ruiz-Martínez, 2014	*	*	(Spouses and caregivers of PD patient)	*		*(Cancer Registry from Department of Health of the Basque Government)	*		4	Poor

[1]Both case-control and cohort studies were conducted.

Supplementary Table 6A. Cancer subtypes and number of PD patients included in each cancer group in the primary analysis.

Cancer group	Cancers included[1]	Number of PD patients
Cancer in general	–	372537
Brain	Brain, malignant brain, benign brain	307706
Colon, rectal, colorectal	Colorectal, colon, rectal	373523
Lung	Lung, lung and bronchus	373415
Melanoma	Malignant melanoma *in situ*, malignant melanoma, malignant melanoma of skin invasive malignant melanoma, unclassified melanoma, melanoma	389257
Oral cavity	Oral cavity, pharynx, lip, oral cavity and pharyngeal, buccal cavity and pharynx, oral cavity/pharynx	76815

Supplementary Table 6B. Cancer subtypes and number of PD patients included in each cancer group in the genetic analysis.

Cancer group	Cancers included[1]	Number of PD patients	
		LRRK2-PD	Idiopathic PD
Cancer in general	–	742	4094
Breast	Breast	742	4094
Brain	Brain, meningioma	632	3551
Colon, rectal, colorectal	Colorectal, colon	711	3962
Haematological	Hematological, leukemia, lymphoma, lymphoma/ haematologic, haematologic, hematologic/lymphoma	742	4094

Supplementary Table 6C. Cancer subtypes and number of PD patients included in each cancer group in the gender analysis.

Cancer group	Cancers included[1]	Number of PD patients	
		Female	Male
Cancer in general	–	107182	139480
Bladder	Bladder, urinary bladder	103885	135652
Colon	Colon, colorectal	97551	128768
Haematological	Leukemia, lymphoma, Hodgkin's lymphoma, non-Hodgkin's lymphoma, myeloid leukemia, lymphatic leukemia, multiple myeloma	107182	139480
Liver	Liver	103885	135652
Lung	Lung	107182	139480
Rectal	Rectum	97551	128768
Renal	Kidney	97551	128768
Stomach	Stomach	103885	135652

[1]These cancer subtypes are quoted as they appeared in the original shortlisted studies.
